# Emotion Regulation, Subjective Well-Being, and Perceived Stress in Daily Life of Geriatric Nurses

**DOI:** 10.3389/fpsyg.2019.01097

**Published:** 2019-05-15

**Authors:** Marko Katana, Christina Röcke, Seth M. Spain, Mathias Allemand

**Affiliations:** ^1^Department of Psychology, University of Zurich, Zurich, Switzerland; ^2^University Research Priority Program “Dynamics of Healthy Aging,” University of Zurich, Zurich, Switzerland; ^3^John Molson School of Business, Concordia University, Montreal, QC, Canada

**Keywords:** emotion regulation, subjective well-being, perceived stress, geriatric nurses, everyday life

## Abstract

This daily diary study examined the within-person coupling between four emotion regulation strategies and both subjective well-being and perceived stress in daily life of geriatric nurses. Participants (*N* = 89) described how they regulated their emotions in terms of cognitive reappraisal and suppression. They also indicated their subjective well-being and level of perceived stress each day over 3 weeks. At the within-person level, cognitive reappraisal intended to increase positive emotions was positively associated with higher subjective well-being and negatively associated with perceived stress. Suppression of the expression of positive emotions was negatively associated with subjective well-being and positively associated with perceived stress. However, cognitive reappraisal intended to down-regulate negative emotions and suppression as a strategy to inhibit the expression of negative emotions were not associated with daily well-being or perceived stress. Off-days were rated as days with higher subjective well-being and lower perceived stress in contrast to working days. At the between-person level, individuals who reported more daily negative affect reported increased suppression of positive emotions, corroborating the within-person findings. Moreover, findings indicated that nurses with more years of experience in the job reported higher subjective well-being and less perceived stress. These results provide insights into important daily emotional processes of geriatric nurses, both at workdays and in their leisure time.

## Introduction

Emotions accompany our daily lives, particularly in contexts that provide a rich array of rewarding or stressful situations. The regulation of these emotions is often a voluntary and conscious act to ensure everyday functioning and to obey social rules. Both experimental and daily diary research have provided interesting insights into regulatory strategies and processes in the daily lives of young, middle-aged, and older adults in relation to subjective well-being (Nezlek and Kuppens, 2008; Brans et al., 2013; Voelkle et al., 2013; Cutuli, 2014; Kalokerinos et al., 2015; Koval et al., 2015; Ong and Zautra, 2015; Brockman et al., 2016; Richardson, 2017; Scheibe et al., 2018). Little is known, however, about emotion regulatory processes as they are experienced in daily life contexts of particular subgroups that are facing interpersonal challenges at work and the association with subjective well-being in those contexts. Providing healthcare to older adults is an emotionally challenging profession requiring specific nurses. In light of the changing demographic landscape, elderly care will increasingly gain importance. Previous work has focused on positive and negative emotional consequences related to caregiving of older adults by informal caregivers such as family members, spouse or adult children (Raschick and Ingersoll-Dayton, 2004; Fortinsky et al., 2007). Little is known about how professional caregivers working with older adults regulate their emotions in their daily lives (both on workdays as well as in their leisure time) and how these regulatory processes are linked with the overall daily subjective well-being and perceived stress.

### Emotion Regulation in Everyday Life

Emotion regulation often involves changes in emotional responding such as increasing, decreasing, or maintaining of positive and negative emotions (Aldao et al., 2010; Webb et al., 2012). These changes may occur on three levels: the kinds of emotions that individuals have, the timing of experiencing their emotions, and how they experience and express these emotions (Koole, 2009; Gross, 2015; Tamir, 2016). Emotion regulation has primarily been conceptualized as an individual difference characteristic that tends to be relatively stable across time and situations (Gross and John, 2003) but differs across age groups (Gross et al., 1997; Urry and Gross, 2010; Hofer et al., 2015; Riediger and Luong, 2015; Scheibe et al., 2015; Röcke et al., 2018). Several emotion regulation strategies have been identified (see for a review, Naragon-Gainey et al., 2017). Two common strategies are *cognitive reappraisal* and *suppression* (Gross, 1998, 2008). Reappraisal is a cognitively oriented strategy that alters the impact of an emotion by either changing the way a situation is constructed or by evaluating an emotional stimulus. Suppression is a response-focused strategy directed toward inhibiting or reducing behaviors associated with emotional responses such as facial expressions, verbal expressions, and gestures. The model of emotion regulation proposed by Gross (1998, 2015) is process-oriented. It suggests that reappraisal is most frequent and most efficient as an antecedent-focused strategy employed prior to or early on in an emotional episode. Following Gross, suppression is a response-focused strategy coming into play during an already ongoing emotional experience (1998, 2015). Cognitive reappraisal and suppression are strategies that aim to alter the intensity or valence of an emotional experience. This is achieved by changing one’s evaluation of emotion-arousing situations or by trying not to show any external signs corresponding to an inner-felt emotion. Both strategies can be applied with regard to positive and negative emotions. In this line of reasoning, reappraisal of positive emotions means changing the way one thinks in order to increase positive emotions. Reappraisal of negative emotions means changing the way one thinks in order to decrease negative emotions. Suppression of positive emotions means inhibiting the expression of positive emotions and suppression of negative emotions means inhibiting the expression of negative emotions (Nezlek and Kuppens, 2008). Typically, an individual’s goal is to enhance positive and to minimize negative emotional experience but there are some exceptions to this pattern (Riediger et al., 2009; Tamir, 2009). For example, the display of positive (or neutral) emotions at the workplace seems particularly important in the context of professional caregiving, as the profession of geriatric nursing requires affective and mental balance (see Mauk, 2010). Most work in applied psychology has focused on the requirement to display positive emotions in spite of the actual felt emotions that an individual may be genuinely experiencing (Grandey, 2003; Scott and Barnes, 2011).

### Emotion Regulation and Subjective Well-Being

A recent meta-analysis of 79 studies has shown that emotional dynamics are associated with well-being (Houben et al., 2015). More specifically, between-person research demonstrates that cognitive reappraisal and suppression are associated with different outcomes (Gross and John, 2003; John and Gross, 2004; Gross, 2015). For example, frequent use of reappraisal has been found to be related with beneficial outcomes such as greater psychological and physical well-being and improved interpersonal functioning (Zapf, 2002; Butler and Randall, 2013). After all, individuals who habitually use reappraisal experience higher subjective well-being because the key function of reappraisal is to diminish negative emotions (Hu et al., 2014). In contrast, frequent use of expressive suppression has been found to be related to more depressive symptoms, diminished well-being and lower interpersonal functioning (Ehring et al., 2010). Research suggests that only reappraisal leads to an enhanced control of emotion (Gross and John, 2003). Individual differences in emotion regulation have also been found to be associated with the temporal dynamics of affect. For example, the affective home base (i.e., intraindividual mean level), variability of affect and attractor strength back to home base have been suggested to be dynamic indicators of emotional experience (Kuppens et al., 2010). In that line of research, a recent study found that suppression was associated with higher emotional inertia (i.e., less variability due to resistance to change) of negative behavior (Koval et al., 2015, 2016).

Apart from differential findings when comparing regulatory strategies, both positive and negative affect also reflect different functions (Cacioppo and Gardner, 1999; Panksepp, 1998; Kim and Hamann, 2007). The seminal model of subjective well-being (Diener et al., 1999) discriminates between an affective component (positive affect and negative affect) and a cognitive component (life satisfaction and domain satisfaction). This distinction could be particularly important in contexts with highly salient display rules pitted against subjective theories about normative emotional experiences. There may be contexts in which the display of either positive or negative emotions is needed. In these cases, discrepancies between these (perceived) expectations and how one actually feels may be particularly unpleasant and dysfunctional. This is the rationale for investigating the valence of emotion regulation strategies at the within-person level. Research has shown that reappraising positive emotions is related to increases in positive affect, self-esteem, and psychological adjustment. Reappraising negative emotions appears to be unrelated to these outcomes (Nezlek and Kuppens, 2008). Another daily diary study consisting of 187 students indicated that days with higher use of cognitive reappraisal were related to days with higher positive affect, and that daily suppression was related to higher negative affect (Brockman et al., 2016). In a similar vein, another experience sampling study has reported within-person results indicating that suppression was associated with higher negative affect and lower positive affect, whereas reappraisal was associated with higher positive affect (Brans et al., 2013). Such differential findings support the distinctive implications of regulating positive emotions versus negative emotions. Our hypotheses follow both theoretical notions of the functions of emotion regulation proposed by Gross (1998, 2015) and previous empirical evidence (e.g., Nezlek and Kuppens, 2008). On the one hand, we expect daily cognitive reappraisal to be positively related to subjective well-being within individuals. On the other hand, we expect daily suppression to be negatively related to subjective well-being within individuals.

• Hypothesis 1: On an average day, more reappraisal is associated with higher subjective well-being.
• Hypothesis 2: On an average day, more suppression is associated with lower subjective well-being.

### Emotion Regulation and Perceived Stress

Everyday life stressors as proxies for context have been extensively studied with regard to subjective well-being (Scott et al., 2013; Wrzus et al., 2015). For example, an experience sampling study with on average of 55 prompts over 3 weeks assessed negative affect, occurrence of stressors, time passed since the stressor occurred and current preoccupation with the occurrence of this stressor (Wrzus et al., 2015). Results showed that generally more recent stressors were associated with lower activating but higher deactivating negative affect. Other studies showed that short-term variability in affect was associated with diurnal rhythm and stressful encounters in everyday life (Steptoe et al., 2011; Piazza et al., 2013). Given the association between subjective well-being and stressors as well as the association between subjective well-being and emotion regulation, perceived stress is an important variable when describing emotional functioning in everyday life. Especially the understudied sample of geriatric nurses reflects an everyday context that requires both a rich opportunity structure and the need for adaptive emotion regulation processes (Duffy et al., 2009; Leiter and Maslach, 2009). Geriatric nurses in their daily work contexts address various physical, psycho-social, cultural and family concerns while promoting health and emphasizing successful aging at the same time (Mauk, 2010). Despite many satisfying and rewarding aspects, the job of professional caregiving can be frustrating and stressful. For example, geriatric nurses often face chronic lack of time, irregular working hours, limited career opportunities, demanding and disruptive behaviors of the persons being cared for, or tensions between caregivers and family members of the patients. Stress and conflicts caused by the demands of caregiving may lead to negative emotions such as anger, anxiety, or depression (Pinquart and Sörensen, 2003; Smith et al., 2011; Richardson, 2017). Therefore, individual factors such as coping skills or the ability to manage daily stress and conflicts are essential individual resources to be considered in the context of caregiving. Previous research on work-life conflict showed that conflicts at work are not independent from subjective well-being experienced during leisure time, suggesting that well-being in the workplace and well-being in leisure time should not necessarily be separated (Knecht et al., 2016).

• Hypothesis 3: On an average day, more reappraisal is associated with lower perceived stress.
• Hypothesis 4: On an average day, more suppression is associated with higher perceived stress.

### The Present Study

This daily diary study focused on how the use of different emotion regulation strategies varies within-person and how these strategies are related to daily subjective well-being and perceived stress. A sample of adults who have an emotionally challenging profession, namely geriatric nursing, have participated in the study over three consecutive weeks. Note that we do not investigate emotion regulation exclusively on workdays but on both workdays and days off work. Therefore, the results can be differentiated between the person’s workday versus off-day average emotion regulation effects on subjective well-being and perceived stress. We also assume that with longer experience in the profession as a nurse, individuals develop emotion regulation strategies for dealing with challenges at work and in their leisure time (Spence Laschinger et al., 2009). To some individuals, nightshifts and the amount of work (full time vs. part time) pose an additional source of stress and decrease subjective well-being (Coffey et al., 1988; Dehring et al., 2018). Therefore, we controlled for non-work days, job experience, number of nightshifts and employment rate in our statistical models.

## Materials and Methods

### Participants and Procedure

This study was conducted in accordance with ethical principles of the Ethics Committee of the Faculty of Arts and Social Sciences of the University of Zurich and in accordance with the Helsinki declaration. Recruitment events were held in four different institutions (i.e., nursing homes) in the area surrounding the city of Zurich and potential participants were provided with detailed information about the project. Moreover, potential participants received a mailing with information about the study via institutional channels. Inclusion criteria were: (a) being a geriatric nurse or a geriatric nurse in training; (b) being employed or in training in one of the four participating institutions; (c) having German language skills. Recruited participants were proportionate to the size of the institution where the events were held. In total, 90 nurses enrolled for the study. One nurse was excluded from the analyses because he or she quit the study after baseline assessment and did not report any daily diary data. No further exclusion criteria were applied. Participants consisted of a convenience sample of geriatric nurses (*N* = 89). The geriatric nurses ranged in age between 17 to 60 years (*M* = 43.48 years, *SD* = 11.25). Seven nurses did not indicate their age. Two nurses were younger than 24 years and were in vocational training. Seventy-seven nurses were female, six were male, and six nurses did not indicate their gender. The unequal gender distribution in this study mirrors the gender distribution in helping professions (Hooker et al., 2016). With respect to marital status, 28 geriatric nurses were married, 39 were single, 16 were divorced or separated, one was widowed, and five did not indicate their marital status.

As we were interested in the within-person processes in daily life, we used a daily diary approach (Nezlek, 2011; Bolger and Laurenceau, 2013). Geriatric nurses provided data on daily emotion regulation, daily subjective well-being, and daily perceived stress using paper-and-pencil diaries at the end of each day for 3 weeks, providing an average of 18.2 days of data (*SD* = 4.0, range = 5–20). In total, we obtained 85% data out of the potential 1,780 observations (89 participants × 20 days). All participants gave their written informed consent prior to study participation. Throughout the study, the geriatric nurses received weekly postal reminders that stressed the importance of adhering to the daily assessment protocol. The nurses received written feedback with general findings at the end of the study.

### Measures

#### Daily Emotion Regulation

Emotion regulation was measured with four items from the Emotion Regulation Questionnaire (ERQ; Gross and John, 2003). As the ERQ is a measure of dispositional emotion regulation strategies, items were reworded slightly to make them appropriate for *daily* administration following the procedure implemented by Nezlek and Kuppens (2008). All items started with: “Thinking back about today, how would you respond to the following question?” Reappraisal was measured with the following two items: “When I wanted to feel a more positive emotion (such as happiness or amusement), I changed what I was thinking about” (*positive reappraisal*); “When I wanted to feel less negative emotion, I changed what I was thinking about” (*negative reappraisal*). Suppression was measured with the following two items: “When I was feeling positive emotions, I was careful not to express them” (*positive suppression*); “When I felt negative emotions (such as sadness, nervousness, or anger), I was careful not to express them” (*negative suppression*). Daily responses were made on a 7-point Likert-type scale ranging from 0 (*not at all characteristic of me*) to 6 (*very characteristic of me*).

#### Daily Subjective Well-Being

Subjective well-being was measured with both emotional and cognitive characteristics (Diener et al., 1999). The emotional facet of subjective well-being was captured by assessing daily positive and negative affect. Affect items were selected to represent both higher and lower arousal affective experience and have been used in previous studies (e.g., Allemand et al., 2012). Daily positive affect was measured with the following items: satisfied, happy, confident, hopeful, active, energetic, joyful, relaxed, and alert. Daily negative affect was measured with the items: disappointed, sad, anxious, worried, sluggish, exhausted, angry, upset, and tired. Participants were asked to rate how strongly they felt each adjective on average during the day. All responses were made on a 7-point Likert-type scale ranging from 0 (*not at all*) to 6 (*extremely*). The within-person reliability of positive and negative affect was *R*c = 0.79 and *R*c = 0.75, respectively, (see, Bolger and Laurenceau, 2013).

To capture the cognitive-evaluative facet of subjective well-being, we used the three items reported in Nezlek and Kuppens (2008). All items started with: “Thinking back about today, how would you respond to the following question?”: “Overall, how positively did you feel about yourself today” (*view of self*); “Thinking of your life in general, how well did things go today” (*view of life in general*); “How optimistic are you about how your life (in general) will be tomorrow” (*optimistic view of the future*). All responses were made on a 7-point Likert-type scale ranging from 0 (*not at all*) to 6 (*extremely*). The within-person reliability of the cognitive well-being scale was *R*c = 0.81.

#### Daily Perceived Stress

Perceived stress was measured with a single-item measure that asked participants to rate the intensity of stress they felt during the day on a 7-point Likert-type scale ranging from 0 (*not at all*) to 6 (*extremely*). Previous research has demonstrated that a single item measure of perceived stress has satisfactory content, criterion and construct validity and can be used for assessing job stressors (Elo et al., 2003; Gilbert and Kelloway, 2014).

#### Control Variables

Accounting for potential third variables in the coupling between emotion regulation and well-being as well as perceived stress, we controlled for whether the daily diary was answered on a workday or an off-day. This was a dichotomous variable, 0 indicating the geriatric nurse was working on that day, 1 indicating the day was an off-day. Moreover, the geriatric nurses indicated how often they worked during nightshift and provided information on their employment rate. Both indications of the number of nightshifts and the employment rate were answered by the nurses with a percentage term. Jobs experience was assessed in terms of how many years a nurse was working in this particular profession. The time variable (i.e., how many days elapsed since the beginning of the study) was used to account for the linear assessment and potential reactivity effects during the study. The applied statistical models also controlled for the person-mean of each daily emotion regulation variable in order to differentiate between the effect of the aggregated mean-levels of emotion regulation and the daily fluctuations around this mean-level (see Bolger and Laurenceau, 2013).

### Statistical Analyses

The statistical analyses focused on day-to-day within-person coupling between emotion regulation, subjective well-being, and perceived stress by using a multilevel model with random intercepts and random slopes. The statistical procedures followed the guidelines described by Bolger and Laurenceau (2013) for repeated observations (level 1) nested within individuals (level 2). The advantage of multilevel modeling analysis is its ability to handle missing data, accounting for the non-independence of the error terms within individuals with repeated measurements and modeling between-person as well as within-person effects simultaneously (Bolger and Laurenceau, 2013). Multilevel modeling analyses were performed in R (R Core Team, 2017) using the lme4 package (Bates et al., 2015) and lmerTest package (Kuznetsova et al., 2017). We ran the analyses in two steps. First, we specified unconditional models for all daily variables, i.e., models with no predictors at either level, to examine the proportion of within-person and between-person variance in the diary data and to ensure that our daily measures of interest had sufficient within-person variability to make within-person analyses feasible. For example, if everyone were stable over the study period, the only variation that would occur would be between-person variation, simply reflecting individual differences in emotion regulation, subjective well-being and perceived stress and no intraindividual covariation with any of the other time-varying variables could be expected. Second, to examine within-person relationships between emotion regulation and subjective well-being as well as perceived stress, we added all independent variables to our models in a stepwise procedure. To account for interindividual and intraindividual variation, we decomposed our daily variables (e.g., PA) into a between-person and a within-person part. The between-person part is the aggregated mean of all observations within an individual. The within-person part is the raw value of each day. This decomposition was applied in order to truly examine the fluctuations from day to day and controlling for the between-person differences (i.e., mean value) at the same time. The between-person variables were centered around the grand-mean and the within-person variables were centered around the person-mean. The time variable depicting the repeated assessment across days was centered at the average day. An estimate of effect sizes can be computed in a similar way as *R*^2^ in multiple regressions and quantifies the variance in the outcome variable explained by all predictor variables in the multilevel model (Peugh, 2010). We report conditional Pseudo-*R*^2^ values representing the proportion of the total variance explained by both fixed and random effects (Nakagawa et al., 2017).

To examine within-person couplings between emotion regulation and subjective well-being as well as perceived stress, we performed multilevel modeling analyses that included all emotion regulation measures as independent variables at the diary level of analysis (level 1: within-person) and the daily subjective well-being measures and daily perceived stress as dependent variables. Following the suggestion of Bolger and Laurenceau (2013), we used person-mean centered independent variables on level 2 and deviation scores for each person from their own mean on level 1. More specifically, for each of the dependent variables, we estimated the following multilevel model (reappraisal of positive and negative emotions, i.e., PR and NR, and suppression of positive and negative emotions, i.e., PS and NS). The within-person variability is modeled with the regression equation:

Level 1:

$$Y_{\text{ij}}\;=\;{\text{β}}_{\text{0j}}\;+\;{\text{β}}_{\text{1j}}(daily\;PR)\;+\;{\text{β}}_{\text{2j}}(daily\;NR)\;+\;{\text{β}}_{\text{3j}}(daily\;PS)\;+\;{\text{β}}_{\text{4}}{}_{\text{j}}(daily\;NS)\;+\;{\text{β}}_{\text{5j}}(off-day)\;+\;{\text{β}}_{\text{6j}}(time)\;+\;r_{\text{ij}}$$

Level 2:

$$\begin{array}{cc} {\text{β}}_{\text{0j}}\;=\; & {\text{γ}}_{\text{00}}\;+\;{\text{γ}}_{\text{01}}(person-mean\;PR)\;+\;{\text{γ}}_{\text{02}}(person-mean\;NR)\;+\\ & {\text{γ}}_{\text{03}}(person-mean\;PS)\;+\;{\text{γ}}_{\text{04}}(person-mean\;NS)\;+\;\\ & {\text{γ}}_{\text{05}}(employment\;rate)\;+\;{\text{γ}}_{\text{06}}(job\;experience)\;+\;\\ & {\text{γ}}_{\text{07}}(nightshift)\;+\;U_{\text{0j}} \end{array}$$

$${\text{β}}_{1j}={\text{γ}}_{10}+u_{1j}$$

$${\text{β}}_{2j}={\text{γ}}_{20}+u_{2j}$$

$${\text{β}}_{3j}={\text{γ}}_{30}+u_{3j}$$

$${\text{β}}_{4j}={\text{γ}}_{40}+u_{4j}$$

$${\text{β}}_{5j}={\text{γ}}_{50}+u_{5j}$$

$${\text{β}}_{6j}={\text{γ}}_{60}+u_{6j}$$

These equations allow for individual variability in the regression coefficients, β_0_ to β_6_. Variability in the regression coefficients allows for individual differences in the intercepts (β_0_) and the six slopes (β_1_ – β_6_). Therefore, different individuals may start at different levels and have different distributions of their slopes (Hoffman, 2007). In addition, the variability in intercepts can be explained by adding different predictors at the between-person level, such as person-mean PR, person-mean NR, person-mean PS, person-mean NS, employment rate, job experience and percentage of nightshift work.

## Results

### Descriptive Statistics

The within-person correlations among the main variables are depicted in Table 1. Moreover, the descriptive analysis showed that the geriatric nurses had on average 17.15 years of experience in their job (*SD* = 10.7) ranging from 1 to 39 years. As can be expected, job experience was positively associated with age (*r* = 0.57, *p* < 0.001). The average percentage of employment rate in this sample was 82.6% (*SD* = 16.36), ranging from part-time nurses working in a 30% contract to full-time nurses. In 55% of the days of the study, the nurses were working. Eighteen percent of the geriatric nurses were working nightshifts. Perceived daily stress was not significantly correlated to adherence to the daily protocol (*r* = -0.11, *p* = 0.31).

**Table 1:** Descriptive statistics and within-person correlations among the main variables.

Variable	1	2	3	4	5	6	7	8
1. Positive affect^a^								
2. Negative affect^a^	-0.63							
3. Cognitive well-being^b^	0.64	-0.56						
4. Perceived stress^a^	-0.45	0.56	-0.43					
5. Positive reappraisal^a^	0.13	-0.11	0.08	-0.06				
6. Negative reappraisal^a^	0.06	-0.02	0.02	0.04	0.57			
7. Positive suppression^a^	-0.20	0.17	-0.18	0.14	0.01	-0.01		
8. Negative suppression^a^	-0.02	0.03	-0.04	0.08	0.18	0.27	0.22	
*M*	3.93	1.54	5.35	1.85	2.99	2.90	1.38	2.39
*SD*	1.00	1.08	1.02	1.71	1.53	1.56	1.50	1.88
ICC	0.46	0.45	0.34	0.32	0.40	0.38	0.49	0.44

*The descriptive statistics are based on *N* = 89. The within-person correlations are based on daily scores nested in individuals and not on aggregated scores. For *N* = 1538 observations, the minimum significant *r* at the 0.05 level is 0.05. ^*a*^0 – 6, ^*b*^1 – 7; ICC = intraclass correlation*.

The intraclass correlations (ICC) were calculated as the between-person variance (level 2) divided by the total variance, i.e., the sum of the between-person (level 2) and within-person variances plus residual variance (level 1). The ICC represents the relative proportion of variance that is between-person with respect to the total variance. The ICC of positive affect was 0.46, of negative affect 0.45, of cognitive well-being 0.32, and of perceived stress 0.32. With regard to the emotion regulation strategies, the ICC of positive reappraisal was 0.40, of negative reappraisal 0.38, of positive suppression 0.49, and of negative suppression 0.44 (Table 1). Thus, more than half of the overall variance was at the daily, within-person level, suggesting that individuals varied around their usual level somewhat more than they differed from each other. Overall, the descriptive results show that the daily measures had sufficient within-person variances to make within-person analyses feasible. Moreover, it should be noted that the means of each measure were sufficiently far from either endpoint. Hence, floor and ceiling effects were no considerations.

### Emotion Regulation Strategies and Subjective Well-Being

Table 2 contains the results of the main multilevel modeling analyses. These results show the within-person coupling between emotion regulation strategies and three subjective well-being indicators, represented in hypotheses 1 and 2. Results with regard to reappraisal strategies showed that the daily reappraisal of positive emotions was negatively related to negative affect (*b* = -0.09, *SE* = 0.03, *p* < 0.01) and positively related to positive affect (*b* = 0.09, *SE* = 0.03, *p* < 0.001) and cognitive well-being (*b* = 0.09, *SE* = 0.03, *p* < 0.01). There was no statistically significant within-person coupling between reappraisal of negative emotions and any subjective well-being indicator (*p* > 0.05). Results with regard to suppression strategies showed that the daily suppression of positive emotions was negatively related to positive affect (*b* = -0.11, *SE* = 0.02, *p* < 0.001) and cognitive well-being (*b* = -0.12, *SE* = 0.02, *p* < 0.001) and positively related to negative affect (*b* = 0.10, *SE* = 0.02, *p* < 0.001). Again, there was no statistically significant within-person coupling between reappraisal of negative emotions and any subjective well-being indicator (*p* > 0.05).

**Table 2:** Within-person coupling between daily emotion regulation and daily well-being measures and perceived stress.

	Positive affect		Negative affect		Cog well-being		Perceived stress	
	*b*	*SE*	*b*	*SE*	*b*	*SE*	*b*	*SE*
**Fixed effects**								
Intercept	3.85*	0.11	1.72*	0.10	5.30*	0.09	2.46*	0.16
Daily positive reappraisal	0.09*	0.03	-0.09*	0.03	0.09*	0.03	-0.09*	0.04
Person-mean positive reappraisal	0.23	0.13	0.04	0.15	0.18	0.12	0.28	0.17
Daily negative reappraisal	-0.01	0.02	0.03	0.02	-0.01	0.03	0.07	0.04
Person-mean negative reappraisal	-0.07	0.14	0.12	0.16	-0.07	0.12	0.01	0.18
Daily positive suppression	-0.11*	0.02	0.10*	0.02	-0.12*	0.02	0.08*	0.03
Person-mean positive suppression	-0.08	0.08	0.24*	0.08	-0.05	0.07	0.02	0.10
Daily negative suppression	0.01	0.01	<0.01	0.02	-0.01	0.02	0.03	0.03
Mean negative suppression	-0.02	0.07	-0.01	0.07	-0.09	0.06	-0.03	0.09
Employment rate	<0.01	0.05	0.05	0.06	<0.01	0.05	0.08	0.07
Job experience	0.02*	0.01	-0.01	0.01	0.02*	0.01	-0.02*	0.01
Off-day	0.21*	0.06	-0.33*	0.04	0.19*	0.06	-1.28*	0.12
Nightshift	-0.02	0.20	0.13	0.22	-0.33	0.18	0.66*	0.26
Time	<0.01	<0.01	<0.01	0.01	<0.01	<0.01	-0.01	0.01
**Random effects**								
Between-person:								
Intercept	0.75*		0.47*		0.41*		1.46*	
Daily positive reappraisal	0.04*		0.03*		0.04*		0.05*	
Daily negative reappraisal	0.01*		<0.01		0.03*		0.04*	
Daily positive suppression	0.02		0.01		0.01		<0.01	
Daily negative suppression	<0.01		<0.01		<0.01*		0.01*	
Off-day	0.19*		0.07*		0.10*		0.89*	
Time	<0.01*		<0.01*		<0.01*		<0.01*	
Within-person:								
Residual	0.34*		0.45*		0.53*		1.15*	
Pseudo-*R*^2^	0.67		0.63		0.51		0.62	

*N = 1456–1477 observations. *SE* represents the standard error of the unstandardized regression coefficients. Pseudo-*R*^*2*^ are the conditional values representing the whole model. ^∗^*p* < 0.05*.

Control variables in terms of person-mean emotion regulation strategies showed no significant associations except for a positive association between the person-mean of suppression of positive emotions and negative affect (*b* = 0.24, *SE* = 0.08, *p* < 0.01). Control variables in terms of work-related third variables in the association between emotion regulation strategies and subjective well-being showed that job experience was positively related to positive affect (*b* = 0.02, *SE* = 0.01, *p* < 0.001) and cognitive well-being (*b* = 0.02, *SE* = 0.01, *p* < 0.001). Off-days were positively related to positive affect (*b* = 0.21, *SE* = 0.06, *p* < 0.001) and cognitive well-being (*b* = 0.19, *SE* = 0.06, *p* < 0.001) and negatively related to negative affect (*b* = -0.33, *SE* = 0.04, *p* < 0.001).

The random effects showed that there were individual differences in the intercepts of all three indicators of subjective well-being; positive affect (var = 0.75, *p* < 0.001), negative affect (var = 0.47, *p* < 0.001), and cognitive well-being (var = 0.41, *p* < 0.001). There were also individual differences in the slopes on positive appraisal for positive affect (var = 0.03, *p* < 0.001), negative affect (var = 0.03, *p* < 0.001), and cognitive well-being (var = 0.04, *p* < 0.001). There were individual differences in slopes on negative reappraisal for positive affect (*var* < 0.01, *p* < 0.001) and cognitive well-being (var = 0.03, *p* < 0.05). There were individual differences in the slopes on negative suppression (var < 0.01, *p* < 0.01) for cognitive well-being. There were individual differences in slopes on off-day for positive affect (var = 0.19, *p* < 0.001), negative affect (var = 0.07, *p* < 0.001), and cognitive well-being (var = 0.10, *p* < 0.001).

### Emotion Regulation Strategies and Perceived Stress

Results with regard to hypotheses 3 and 4 about the within-person coupling between perceived stress and emotion regulation strategies showed the following results (Table 2). Daily reappraisal of positive emotions was negatively related to perceived stress (*b* = -0.09, *SE* = 0.04, *p* < 0.05). Daily suppression of positive emotions was positively related to perceived stress (*b* = 0.08, *SE* = 0.03, *p* < 0.01). However, daily reappraisal of negative emotions and daily suppression of negative emotions were not related to perceived stress (*p* > 0.05).

Control variables in terms of person-mean emotion regulation strategies showed no significant associations with perceived stress (*p* > 0.05). Control variables in terms of work-related third variables in the association between emotion regulation strategies and perceives stress showed that job experience was negatively related to perceived stress (*b* = -0.02, *SE* = 0.01, *p* < 0.05). Off-days were also negatively related to perceived stress (*b* = -1.28, *SE* = 0.12, *p* < 0.001). Amount of nightshift work was positively associated with perceived stress (*b* = 0.66, *SE* = 0.26, *p* < 0.001). The random effects showed that there were individual differences in the intercepts of perceived stress (var = 1.46, *p* < 0.001). There were also individual differences in the slopes on positive appraisal for perceived stress (var = 0.05, *p* < 0.001) and in the slopes on negative reappraisal for perceived stress (var = 0.04, *p* < 0.01). There were individual differences in the slopes of daily negative suppression (var = 0.01, *p* < 0.01) and off-days (var = 0.89, *p* < 0.01) on perceived stress.

## Discussion

This study has three main contributions. First, previous work applying a within-person perspective on regulatory processes has been restricted to samples of college students (Nezlek and Kuppens, 2008; Brans et al., 2013; Richardson, 2017). Underlying motivational processes may differ between such student groups and the current sample of professional caregivers. For example, in the daily lives of students, there are certain display rules in place such that consider loud outbursts of emotion during class to be most often inappropriate. Most likely, however, students do not have as strong a motivation or need to regulate their feelings as professional caregivers who regularly face more extreme emotionally relevant events and interactions and more salient display rules. Second, these results extended previous results by looking at different indicators of subjective well-being (i.e., positive affect, negative affect, cognitive well-being) and differentiating between up-regulating and down-regulating emotions in a unique sample of employees. Especially in the profession of caregivers, everyday life emotion regulation strategies are key to maintaining well-being and preventing stress. Third, this research extended previous findings by showing a robust pattern of coupling between reappraisal of positive emotions and subjective well-being as well as coupling between suppression of positive emotions and subjective well-being, not only on the between-person level but also on the within-person level.

### Regulation of Positive Emotions Relates to Subjective Well-Being

This study found meaningful within-person coupling between emotion regulation strategies and subjective well-being. On the within-person level, the use of cognitive reappraisal to increase or up-regulate positive emotions was associated with increased well-being. Suppression to inhibit the expression of positive emotions was associated with decreased well-being. At the between-person level, some of the within-person patterns were mimicked: nurses who suppressed the expression of positive emotions were also those with higher negative affect. However, it is unclear whether these patterns also hold for long-term consequences. It has yet to be examined whether suppression of emotions might be beneficial in the long-term for other outcomes that are indirectly associated with subjective well-being. On the one hand, for geriatric nurses hiding emotions in certain situations might add to the perceived friendliness by patients and family or could be favorable for job promotions and salary. On the other hand, hiding emotions might cause a lack of perceived authenticity by patients or family members therefore endanger long-term well-being even more. These results have also shown that not only emotion regulation strategies but also job experience was associated with indicators of daily well-being. Since job experience correlates with age, a potential explanation could be that nurses with advanced age and job experiences have learned how to use more efficient emotion regulation strategies in their daily lives (Blanchard-Fields, 2007). These results also mirror prior findings with regard to higher psychological well-being of employees with advanced job experience (Avey et al., 2010; Kooij et al., 2013). Moreover, off-days were associated with higher daily positive affect and higher cognitive well-being. In contrast, off-days were associated with less negative affect and less perceives stress. These results underline previous research on the impact of work on subjective well-being. For example, it has been reported that high workload has a negative impact on work detachment and that low detachment from work has in turn a negative association with well-being (Sonnentag and Bayer, 2005).

### Regulation of Positive Emotions Relates to Perceived Stress

We also found interesting results with respect to daily emotion regulation and perceived stress. More than two-thirds (68%) of the overall variance of perceived stress was at the daily within-person-level, suggesting that caregivers fluctuated around their usual level of perceived stress more than they differed from each other. These results showed meaningful within-person coupling between and emotion regulation strategies and perceived stress. Days on which individuals applied more cognitive appraisal of positive emotions were also days on which individuals reported less perceived stress. Days on which individuals applied suppression of positive emotions were also days on which individuals reported more perceived stress. Not surprisingly, exposure to daily stressors can be associated with a wide range of negative outcomes including decreased well-being and increased social and health problems (Lazarus and Folkman, 1984; Richardson, 2017). However, not all individuals who are exposed to high levels of stressors develop negative outcomes. In fact, a considerable number of individuals is able to remain largely unaffected by their daily stress at work. These results have shown that off-days are related with less perceived stress. This is in line with previous research that showed that most middle-aged adults perceive a vast amount of their everyday life stress at their workplace rather than in their home life (Almeida et al., 2002). The emotional reactions involved in stress entail emotional regulation. When caregivers encounter stressful events in their daily life, emotion regulation enables them to manage their emotions. Geriatric caregiving has received attention in the aging literature mainly with a focus on informal caregivers such as family members rather than formal caregivers such as geriatric nurses. Family members are likely to be more or at least differently emotionally invested in the care recipient (“client”) compared to formal caregivers. They might therefore display different responses and face different emotion regulation experiences than professionals. The latter could be considered experts in both the primary caregiving activities (e.g., assistance with activities of daily life), but also in the related task of regulating the emotions that come with the job. In addition, most research using working samples to study emotion regulation in professional contexts has focused on a different class of jobs. Typically, customer service jobs or entertainment sector jobs have been examined, which are characterized by different and very specific rules regarding emotion regulation and particularly emotion expression (e.g., cheerleaders in Beal et al., 2006; administrative staff in Grandey, 2003). Hence, the present study served as an initial step to understand within-person processes in particularly taxing situations such as the working environment of geriatric nurses.

### Type of Emotion Regulation Strategy Matters

The overall picture suggests that the type of regulated emotion and the type of regulation strategy matters for subjective well-being and perceived stress. It seems that up-regulating positive emotions in contrast to down-regulating negative emotions is differently associated with indicators of subjective well-being and perceived stress. Similar results were previously reported in student samples (Nezlek and Kuppens, 2008; Brans et al., 2013; Brockman et al., 2016; Richardson, 2017). In line with these studies, we found that up-regulation through positive reappraisal was beneficial, whereas regulation by positive suppression was not (John and Gross, 2004). The differential pattern for regulating positive and negative emotions (Kim and Hamann, 2007) follows other work showing that positive affect is not the opposite of negative affect (Lucas et al., 1996; Diener et al., 1999) and supports the importance of considering the valence of emotions in a differential manner. The experience of positive emotions is important at the workplace because it helps employees obtain favorable outcomes such as greater task activity, higher achievement, and higher quality social interactions (Staw et al., 1994; Lyubomirsky et al., 2005). Moreover, according to Fredrickson’s (1998) broaden-and-build theory, positive emotions can broaden individual’s thought-action-repertoire leading to enduring personal resources, which, in turn, may facilitate behavioral flexibility and well-being (Fredrickson, 2004). To that end, the use of emotion regulation strategies with the goal of up-regulating positive emotions is particularly important in the context of professional caregiving to older adults since it may be helpful to maintain well-being. An alternative explanation for no associations between emotion strategies of negative emotions in contrast to emotion strategies of positive emotions could be due to the fact that the sample consisted of healthy individuals. Therefore, they might show only little fluctuation in negative affect in contrast to the fluctuations of positive affect and cognitive well-being. It may well be that in some subgroups, for example clinically ill patients, reappraisal of negative emotions and suppression of negative emotions might, in some cases, be beneficial for their subjective well-being (e.g., Henry et al., 2008; Roberton et al., 2012). Future research is needed to test everyday life emotion regulation strategies in different samples including clinical samples.

### Implications

The present findings have several practical implications. First, knowledge about the emotion regulation strategies that professional caregivers use to influence which emotions they have, when they have them, and how they experience and express them (Gross, 1998, 2015), may be key to a better understanding of subjective well-being in geriatric nurses, employee satisfaction, and employee engagement. Emotion regulation skills could be facilitated by specific training. In this daily diary study, we demonstrated that emotion regulation strategies were related to different indicators of subjective well-being and perceived stress. Second, a focus on daily within-person processes clearly complements between-person differences research that compares individuals with higher versus lower scores in the constructs of interest (Mroczek et al., 2003). Both approaches are important. The between-person sources of variance reflect human individuality. Information about individuality may inform individually designed tools or interventions to help caregivers deal adaptively with their emotions. However, as demonstrated in this study, individuals also vary substantially within themselves from day to day. This within-person variation may give important information about the malleability and variability of states and processes of individuals’ lives.

### Limitations and Conclusion

Several limitations should be noted. First, we only focused on two commonly used classes of emotion regulation strategies (Gross, 1998). Future research might include more diverse strategies such as problem solving, disengagement, distraction, rumination, or relaxation (Parkinson and Totterdell, 1999; Trougakos et al., 2008; Naragon-Gainey et al., 2017). Findings from an experience sampling study have shown that distraction is one of the most used emotion regulation strategy in everyday life, and that different types of emotion regulation strategies are used simultaneously (Brans et al., 2013). Second, we relied upon end-of-day reports regarding daily emotion regulation, subjective well-being, and perceived stress. As such, these reports were somewhat retrospective and may have been biased. For example, it is possible that such reports reflect the strongest feelings individuals had during a day, how they were feeling at the end of the day or how they were feeling when they provided the ratings (e.g., Röcke et al., 2011). Future research might include random multiple momentary assessments throughout the day (Wrzus and Mehl, 2015) to draw a more concrete picture of emotion regulation dynamics that are highly context dependent (Aldao, 2013; Kuppens and Verduyn, 2015; Röcke et al., 2018). This would allow to apply a more personalized approach when studying individual emotion regulation processes (Doré et al., 2016). Third, we used paper-and-pencil diaries, which do not offer objective time stamp information on when the diary was actually filled in. However, there is reason to believe that participants agreeing to participate in such a study do provide data that is very comparable to studies using electronic diaries (Green et al., 2006). Fourth, this research examined the everyday lives of geriatric nurses and did not distinguish between work days and off-days. However, we controlled for whether the nurse was on- or off-work. This gives us the person’s workday versus off-day average emotion regulation effects, with whether it’s an on- or off-day controlling for the persons’ average level of workday versus off-day subjective wellbeing, or perceived stress. Finally, to reduce participants’ burden, we used very short daily measures. Our findings may thus be limited by the use of single-item measures. Future research might examine other facets of these complex constructs. For example, it would be interesting to test the coupling between emotion regulation and subjective well-being using other conceptualizations of well-being such as psychological well-being with aspects like environmental mastery (Ryff and Keyes, 1995). Environmental mastery refers to the ability to control and manage one’s environment and one’s efficacy in choosing environments that suit one’s goals and needs. Research at the between-person level found that individuals who habitually use cognitive reappraisal showed more environmental mastery, whereas suppression was correlated negatively with the ability to control and manage the environment (Gross and John, 2003). However, it is unclear whether these associations also hold at the within-person level over time in the context of caregiving.

## Conclusion

In conclusion, this study provides valuable insights into important emotional processes in the daily life of geriatric nurses. In particular, the results indicated that regulation through reappraisal with the goal of up-regulating positive emotions was beneficial, whereas regulation by suppressing the expression of positive emotions was not. In other words, the ability to effectively deal with emotions assists professional caregivers in managing occupational stress and maintaining subjective well-being both during workdays and in their leisure time.

## Author Contributions

MA contributed to the conception and design of the study. MK analyzed the data and drafted the manuscript together with MA, CR, and SS. All authors approved the final version of the manuscript.

## Conflict of Interest Statement

The authors declare that the research was conducted in the absence of any commercial or financial relationships that could be construed as a potential conflict of interest.
